# Integrating enzyme fermentation in lignocellulosic ethanol production: life-cycle assessment and techno-economic analysis

**DOI:** 10.1186/s13068-017-0733-0

**Published:** 2017-02-23

**Authors:** Johanna Olofsson, Zsolt Barta, Pål Börjesson, Ola Wallberg

**Affiliations:** 1 Division of Environmental and Energy Systems Studies, Department of Technology and Society, Lund University, John Ericssons väg 1, 22363 Lund, Sweden; 2 Department of Applied Biotechnology and Food Science, Faculty of Chemical Technology and Biotechnology, Budapest University of Technology and Economics, Muegyetem rkp. 3, Budapest, 1111 Hungary; 3 Department of Chemical Engineering, Lund University, Naturvetarvägen 14, 22362 Lund, Sweden

**Keywords:** LCA, Bioethanol, Second generation (2G), Cellulases, Process economics, On-site, Off-site, Greenhouse gases

## Abstract

**Background:**

Cellulase enzymes have been reported to contribute with a significant share of the total costs and greenhouse gas emissions of lignocellulosic ethanol production today. A potential future alternative to purchasing enzymes from an off-site manufacturer is to integrate enzyme and ethanol production, using microorganisms and part of the lignocellulosic material as feedstock for enzymes. This study modelled two such integrated process designs for ethanol from logging residues from spruce production, and compared it to an off-site case based on existing data regarding purchased enzymes. Greenhouse gas emissions and primary energy balances were studied in a life-cycle assessment, and cost performance in a techno-economic analysis.

**Results:**

The base case scenario suggests that greenhouse gas emissions per MJ of ethanol could be significantly lower in the integrated cases than in the off-site case. However, the difference between the integrated and off-site cases is reduced with alternative assumptions regarding enzyme dosage and the environmental impact of the purchased enzymes. The comparison of primary energy balances did not show any significant difference between the cases. The minimum ethanol selling price, to reach break-even costs, was from 0.568 to 0.622 EUR L^−1^ for the integrated cases, as compared to 0.581 EUR L^−1^ for the off-site case.

**Conclusions:**

An integrated process design could reduce greenhouse gas emissions from lignocellulose-based ethanol production, and the cost of an integrated process could be comparable to purchasing enzymes produced off-site. This study focused on the environmental and economic assessment of an integrated process, and in order to strengthen the comparison to the off-site case, more detailed and updated data regarding industrial off-site enzyme production are especially important.

**Electronic supplementary material:**

The online version of this article (doi:10.1186/s13068-017-0733-0) contains supplementary material, which is available to authorized users.

## Background

With second generation biofuels comes an increased potential for bioenergy worldwide as well as new challenges related to efficient conversion of woody biomass and environmental sustainability. The use of residual lignocellulosic material as feedstock for biofuels has been suggested as a way to avoid potentially negative effects of land use change and competition with human food production [1, 2], which has previously generated concern and debate. Spruce is the most abundant wood species in Sweden, and in several studies it has been shown to be a suitable raw material for bioethanol production [3–6]. The cost and environmental impact of lignocellulosic bioethanol, most notably in terms of greenhouse gases (GHG) emitted, have been assessed in various studies giving results with significant variations [7–23].

Life-cycle assessment (LCA) has become a widespread tool for performing such analyses of the environmental performance of a product by mapping the resource use and emissions related to its life cycle. Thus, LCA is a potential tool for comparing and analysing different pathways for lignocellulosic ethanol as well as finding hot spots for future improvements. Several LCA studies of lignocellulosic ethanol have found the production of enzymes to contribute significantly to environmental impacts, including GHG emissions [7–12]. Enzymes have also been identified as an important factor in total ethanol production costs. Liu et al. [13] calculated costs up to 0.82 EUR L^−1^ ethanol, depending on the price of cellulase enzymes used in enzymatic hydrolysis. Earlier studies reported production costs from approximately 0.10 to 0.59 EUR L^−1^ ethanol, depending on the choice of process design and the assumptions used in the studies [14–23]. The cost of cellulases not only represents a significant part in the overall production costs in current systems, but it is also one of the most uncertain parameters in the evaluations [16], and many have used assumptions based on future prices for manufactured enzyme products [14, 19, 20, 24–28]. Furthermore, the lack of transparency and the difference in the way enzyme dosages are reported hinder comparisons between studies [29].

A potential alternative to purchasing manufactured enzymes is an integrated process of enzyme and ethanol production. By using whole, crude fermentation broths containing fungal cells and substrate residues, the processes of cell removal, enzyme concentration and purification steps could be avoided. Hypercellulolytic mutants of *Trichoderma reesei*, the most widely used fungus for cellulase production, have been reported to grow well and secrete large amounts of cellulolytic enzymes on steam-pretreated spruce [30, 31] and could thus be assessed for an integrated process design [32]. Integrated production entails using part of the wood feedstock for growing *T. reesei*, reducing the fraction of wood available for ethanol conversion, but using the whole fermentation broth could also improve saccharification and ethanol yields due to the effect of mycelium-bound enzymes [33–36]. Some LCA studies have taken steps towards investigating the potential environmental benefits of co-locating and partly integrating enzyme production with ethanol conversion [10–12], but the full potential of an integrated process approach, using part of the lignocellulosic feedstock for enzyme production, has only recently been addressed [37, 38]. Janssen et al. [37] showed that an integrated process could potentially halve GHG emissions from high-gravity ethanol production and significantly improve the performance in other environmental impact categories. In terms of economics, several studies presume that on-site or integrated production of enzymes on cheap lignocellulosic raw materials will be desirable to reduce ethanol costs [13, 21, 29, 39–44].

The aim of the present study was to investigate GHG emissions, primary energy use and the production cost for ethanol made from spruce logging residues in Sweden, using two different process designs for integrated cellulase enzyme and ethanol production. Integrated enzyme production in a full-scale bioethanol plant was modelled together with the whole ethanol production process using the commercial software Aspen Plus. GHG emissions and primary energy balances were assessed in a life-cycle perspective using two standardized calculation frameworks to illustrate the sensitivity of the results to different assumptions made in the LCA method. Ethanol production cost was assessed as minimum ethanol selling price (MESP), meaning the ethanol price at the break-even point where the annual cost and the annual income are equal, as also the annual costs and revenues. The results for the integrated process designs were compared to an off-site case, where enzymes were assumed to be purchased from an off-site facility. The enzyme production in the off-site case was based on existing data for current industrial production reported in previous LCAs of lignocellulosic ethanol [7, 9], and potential improvements and uncertainties regarding GHG emissions, energy and cost performance of off-site enzyme production were assessed in sensitivity analyses. The focus in these sensitivity analyses was on critical factors affecting the comparison between the modelled integrated cases developed in this paper and the off-site case.

## Methods

### Process description

The model of bioethanol production is based on previously published research by Barta et al. [32] and will be introduced here only briefly. For the integrated cases, the entire ethanol plant with integrated enzyme production was modelled. For the off-site case, the ethanol plant was modelled without the external enzyme production facility. The purchased enzymes are added to the modelled ethanol plant according to available information and data regarding industrial enzyme production today. Due to the aggregated nature of such information and data presented in the existing scientific literature, it was not possible to analyse off-site enzyme production in the same level of detail as the modelled integrated production. Thus, there is a difference in the assessment performed in this paper between the cases, where the integrated cases are analysed in more detail than the off-site case.

**Fig. 1:**
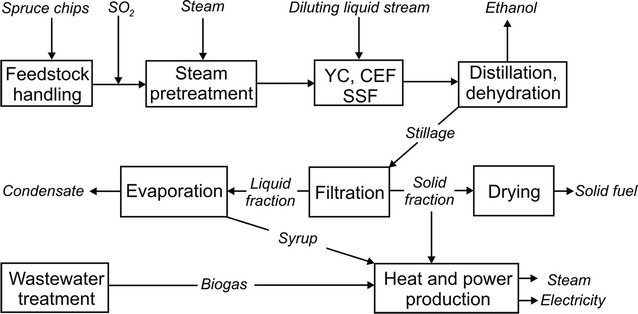
Overall process design for the proposed ethanol plant. In the reference case, there was no integrated enzyme production, instead the enzymes were purchased from an off-site facility. *CEF* cellulase enzyme fermentation, *YC* yeast cultivation, *SSF* simultaneous saccharification and fermentation [32]

The dry spruce chips were assumed to contain 37.9% glucan, 9.9% mannan, 1.8% galactan, 4.3% xylan, 1.3% arabinan, and 28.0% lignin [32]. Cellulases were assumed to be produced using a mutant of *T. reesei*, employing the whole crude fermentation broth of the fungus in the saccharification step. Figure 1 shows the process schematically. A pretreated liquid fraction and a pretreated liquid fraction supplemented with molasses were evaluated as feed for integrated enzyme production. As reference case, ethanol production without integrated enzyme production was modelled, and enzymes were assumed to be purchased from an external enzyme production facility. The latter is the off-site case. In all the scenarios, the conversion factors for some reactions were the following [32]: (1) in steam pretreatment glucan to glucose 0.185, glucan to hydroxymethylfurfural 0.007, xylan to xylose 0.792, xylan to furfural 0.083, water-insoluble lignin to water-soluble lignin 0.037 and (2) in SSF (simultaneous saccharification and fermentation) glucan to glucose 0.91, xylan to xylose 0.8, glucose to ethanol 0.92, glucose to glycerol 0.035. Further details regarding the ethanol production design can be found in the Additional files 1, 2, and in [32].

#### Integrated enzyme fermentation

**Fig. 2:**
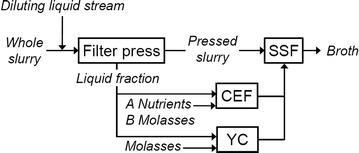
Schematic flow sheet of cellulase enzyme fermentation (CEF), yeast cultivation (YC), simultaneous saccharification and fermentation (SSF). Based on [32]. The configurations A and B differ in carbon source as indicated by the addition of nutrients for case A, and molasses for case B. All nutrients and chemicals included are listed in mass balances in the Additional file 1

Two configurations, denoted A and B, were investigated in the model of integrated enzyme fermentation (Fig. 2). They differed in the carbon source: in configuration A, part of the liquid fraction of the diluted slurry was used, while in configuration B the liquid fraction was supplemented with molasses to increase the sugar content. The enzyme dosage of simultaneous saccharification and fermentation was ten filter paper units (FPU) g^−1^ water-insoluble solid.

Assumptions regarding enzyme dosage in the integrated cases were based on confirmed laboratory-scale results. These results were based on an integrated process setup, and thus impacts on ethanol yields from diversion of part of the hydrolysate for growing *T. reesei*, and the toxicity of the hydrolysate in the cellulase enzyme fermentation, are taken into account (for further discussion of these parameters, see Barta et al. [32]). To illustrate a future scenario, two cases A+ and B+ were included, which use the same setup as A and B, respectively, but with increased enzyme activity. In the cases annotated by “+”, the specific activity of the soluble proteins was enhanced 1.5-fold, resulting in an increase of 50% in the productivity in terms of enzyme activity, while protein and mycelium yields remained the same.

#### Off-site enzyme production

In the off-site case, enzymes were assumed to be purchased from an external production facility and added directly to the SSF in the ethanol plant model. In general, the steps in off-site enzyme production are as follows: (i) production by microorganisms using inputs of carbohydrates, protein, mineral salts and vitamins followed by (ii) the recovery of the enzyme liquor and (iii) formulation of the enzyme product [45]. For the purpose of this study, the important differences between off-site and integrated enzyme production are the treatment steps in (ii) and (iii) applied to refine and stabilize the enzymes intended for use elsewhere (see e.g. [12]), as well as feedstock material. However, since no cost breakdown is available for the current industrial production of enzymes, and environmental data based on industrial scale production are aggregated in the existing scientific literature [see e.g. 7, 9, 45], no detailed data for the different processes of off-site enzyme production can be presented, and therefore off-site enzyme production is analysed in less detail.

The enzyme preparation purchased in the off-site case was based on existing data for the commercially available cellulase cocktail Cellic CTec3 from Novozymes A/S. Assuming 213 FPU mL^−1^ for the cocktail [46, 47] and a density of 1.1 g mL^−1^ (valid for CTec2, the predecessor product) [48], enzyme dosage was calculated to be 30.4 g enzyme cocktail kg^−1^ DM (dry matter) wood. This dosage is used for the base case scenario, but as it is a crucial factor for the resulting costs and GHG emissions, alternative assumptions were tested in sensitivity analyses (see “Alternative enzyme data for sensitivity analyses” section).

### Techno-economic analysis

The model in Barta et al. [32] was updated mainly regarding capital and enzyme costs. Mass and energy balances were solved using the commercial flow sheeting program Aspen Plus V8.0 (Aspen Technology, Inc., Cambridge, MA). The important results of mass and energy balances used as inputs for the life-cycle assessment are included in the Additional file 1 for all the modelled cases of ethanol production. Fixed capital investment (FCI) costs were estimated either with Aspen Process Economic Analyzer V8.0 (Aspen Technology, Inc.) or from vendor quotation (see Additional file 3). To obtain the annual FCI, an annuity factor of 0.11 was used, corresponding to a depreciation period of 15 years and an interest rate of 7%. Working capital investment (WCI) was calculated according to the recommendations in the literature [49]. Annual WCI was calculated by multiplying the WCI by the interest rate.

All costs were calculated in Swedish kronor (SEK) but are presented here in Euros (EUR, 1 US$ ≈ 1.1 EUR, 1 EUR ≈ 9.3 SEK). In the off-site case, the purchase price of enzyme was 3.55 EUR per million FPU, which was obtained by updating the estimate of a previous study [32]. Purchase prices of raw material, nutrients, chemicals, and utilities, costs of labour, insurance, maintenance and selling prices of co-products are found in the Additional file 1, with further economic data presented in the Additional file 3.

**Fig. 3:**
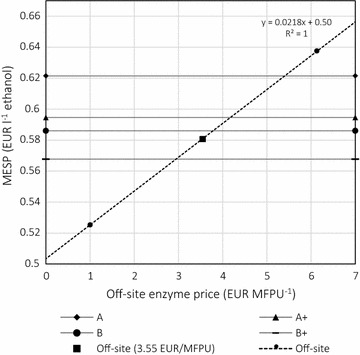
Minimum ethanol selling price (MESP), with off-site MESP as a function of enzyme price. Carbon source A: pretreated liquid fraction, B: pretreated liquid fraction and molasses; +: 1.5-fold specific activity; off-site: reference case with purchased enzyme preparation. The enzyme dosage of simultaneous saccharification and fermentation was 10 FPU g^−1^ water-insoluble solids. The price of enzyme per MFPU can be converted to price per kg of enzyme preparation by multiplying it by a factor of 0.194

Focusing on the comparison between off-site and integrated enzyme production, MESP is calculated in the economic analysis. As the enzyme price is uncertain to a great extent, sensitivity analysis of MESP was performed in the off-site case: MESP was plotted as a function of enzyme price in EUR MFPU^−1^ (Fig. 3). Using the equation of the fitted curve (Fig. 3), the MESP of the off-site case can easily be adjusted to other enzyme prices and compared to integrated cases at any enzyme price.

Annual cash flows show the difference between the annual cost and annual income, and illustrate the distribution of costs and revenues for the ethanol production systems studied. As an illustration, the annual cash flows were calculated using an ethanol selling price of 0.59 EUR L^−1^ (5.5 SEK L^−1^, based on [32]). The results can be found in the Additional file 1.

### Life-cycle assessment

Two calculation approaches for LCA were applied: one following the standardized methodology of ISO 14040 and 14044 [50, 51] and the other following the method presented in the EU renewable energy directive (RED) [52]. Where the ISO method offers a frame and structure for LCA with recommendations regarding method considerations, the RED method goes further by stating how the environmental impact in terms of GHG emissions from biofuel systems is to be calculated [52]. The RED method is designated for calculations of GHG emissions, but for the purpose of this study, RED method assumptions are also applied to calculate primary energy efficiency. Both methods were applied in a well-to-gate analysis, meaning that the environmental life cycle of ethanol was followed from cradle to factory gate.

LCA results for biofuels have proved to be generally affected by methodological choices such as the allocation procedure and the handling of different co-products [53–56]. Both ISO and RED methods call for sensitivity analyses in which the sensitivity of the results to changes of different parameters is tested, and thus applying two calculation approaches can in itself serve as this analysis. As the focus of this study was primarily on the assessment of new integrated process designs, and secondly on a comparison to an off-site case with purchased enzymes, the main objects for scrutiny in the sensitivity analyses were the input data and the assumptions made for enzyme production and utilization. As a result, choices regarding system expansion and crediting co-products in the ISO method are discussed only briefly. Furthermore, this study cannot answer questions concerning an optimized, overall process design for a lignocellulosic ethanol biorefinery, but focuses on one aspect of such an environmentally sustainable and commercially viable design.

#### Functional unit and system boundaries

According to RED, the functional unit (FU) to which environmental impact is related is 1 MJ (LHV) of fuel [52]. For the purpose of comparison, the FU was 1 MJ bioethanol (LHV) in both ISO and RED calculations. GHG performance was calculated as global warming potential (GWP) with a 100-year time-frame. Emissions of CO_2_, CH_4_ and N_2_O were taken into account, where 1 g of CH_4_ and N_2_O was regarded as 34 and 298 g CO_2_-eq., respectively [57].

In the RED methodology, wastes and agricultural crop residues used as feedstock in biofuel production are free from GHG emissions from activities prior to their collection [52]. RED also states that CO_2_ uptake during cultivation and CO_2_ emissions from combustion of biofuels are to be excluded from the calculations. For the ISO method, CO_2_ uptake during biomass growth was assumed to be equal to CO_2_ emissions stemming from the biomass in the fermentation process and from ethanol combustion. No environmental impact from forestry was allocated to forest residues, other than from the collection and transportation of residues. However, the collection of residues may affect soil carbon content as biomass is removed from the forest (see e.g. [58]). Nutrient balances can also be affected negatively, though ash recovery can reduce such issues [59]. These aspects were excluded from the present calculations due to the study focus here being on the potential for integrated enzyme production, but effects on soil carbon are included in a sensitivity analysis.

#### Multi-functionality and allocation

The ethanol systems studied are multi-functional and deliver electricity and lignin solid fuel as co-products. According to the ISO order of priority, expansion of the system to include co-products is preferred prior to the allocation of environmental burden based on physical or economic relationships [51]. For the ISO method we applied substitution, a form of system expansion where co-products are assumed to substitute corresponding products. Electricity was assumed to be delivered to the grid, replacing the regional electricity mix. Avoided environmental impacts from substituted products were credited to ethanol.

In the RED method, environmental impacts are allocated to co-products based on lower heating value (LHV) [52]. The RED guidelines state that electricity is regarded as a co-product if generated from by-products or waste at the plant, and in other scenarios it is assumed to substitute grid electricity [52]. Here electricity was regarded as co-product for RED calculations.

#### Inventory

Table 1 shows GHG and primary energy data for nutrients, chemicals, enzymes, electricity mixes and fuels included in the assessment.

**Table 1:** LCA data for chemicals, nutrients and enzymes

Input	kg CO_2_-eq. kg^−1^	MJ primary energy kg^−1^ (for production)	Source
Sulphur dioxide SO_2_	0.42	7.8	[7, 60]
Ammonia NH_3_	3.23	11.1	[61]
Phosphoric acid H_3_PO_4_	1.36	5.52	[61]
Antifoam	1.33	24.4	Average based on [62] and [63]
Diammonium phosphate (NH_4_)_2_HPO_4_	0.87	8.19	[61]
Magnesium sulphate MgSO_4_	0.308	1.1	[64]
Molasses	0.142	0.57	[7, 65]
Soybean oil meal	0.8	5.95	[65]
Ammonium sulphate (NH_4_)_2_SO_4_	2.6	10.4	[61] (primary energy based on general data for N-fertilizer)
Monopotassium phosphate KH_2_PO4	0.287	26	[66]
Iron(II) sulphate heptahydrate FeSO_4_*7H_2_O	0.093	1.13	Data for FeSO_4_ [60]
Enzymes	5.5	69^a^	Personal communication with Kløverpris, J. H., Novozymes A/S, February 2015. Estimation based on [7]
	g CO_2_-eq. MJ^−1^	Primary energy factor	
Swedish electricity mix	10.1	2.1	[67]
Natural gas-based electricity	124	1.9	[67, 68]
Hard coal	106	1.15	[67]

^a^Carbon footprint was re-evaluated by Novozymes A/S from 8 to 5.5 kg CO_2_-eq. kg^−1^ enzyme cocktail, a reduction by 31%. Fossil energy use was previously 100 MJ kg^−1^ cocktail, from which the estimate here is reduced by 31%

##### Collection and transportation of feedstock

We assumed that the forest residues were collected as loose logging residues (tops and branches) after final felling of spruce stands. Collection, forwarding, loading, unloading, comminution and transport of feedstock were assumed to be conducted as in Lindholm et al. [58, 69]. Feedstock was collected as loose residues in northern Sweden where transportation distance was estimated, on average, to be 138 km.

Based on LHV of 19.2 MJ kg^−1^ DM for forest residues, GHG emissions related to harvest and transport activities were 65 and 42 g CO_2_-eq. kg^−1^ DM collected, respectively [58]. Energy input was 0.25 MJ kg^−1^ DM for harvest and 0.25 MJ kg^−1^ DM for transport [69].

##### Enzyme and nutrients

For integrated cases, primary energy input and GHG emissions for chemicals and nutrients shown in Table 1 were included in the calculations. Regarding the energy content of the products, only the energy content of molasses (13.6 MJ kg^−1^ DM based on Aspen modelling) and purchased enzymes (assuming 10% protein concentration and 11.2 MJ kg^−1^ protein, based on Aspen modelling) were taken into account.

As described in the previous sections, the off-site production of purchased enzymes was not modelled, in contrast to the integrated enzyme production cases, and thus life-cycle data are based on the existing scientific literature and other available information. For the off-site case, GHG emission data for the Novozymes A/S Cellic CTec3 enzyme product were based on previously used data [7, 9] that were updated based on personal communication with Novozymes A/S (personal communication with Kløverpris, J. H., Novozymes A/S, February 2015). The data refer to aggregated GHG emissions from the production at the company site in North Carolina, United States, which amount to 5.5 kg CO_2_-eq. kg^−1^ product. No further breakdown of GHG emissions was available but alternative data for production of the purchased enzymes were tested in the sensitivity analyses, and thus making possible more thorough comparisons of the integrated and off-site cases.

Previously released data from Novozymes A/S contained information on the aggregated input of fossil primary energy in the production process. As presented in [7], fossil primary energy input was 100 MJ kg^−1^ formulated product based on aggregated data from 2012, where the corresponding data for GHG emissions were 8 kg CO_2_-eq. kg^−1^ product. For the purpose of this study, assumptions regarding primary energy input in off-site enzyme production were necessary:

i. The source of electricity in the off-site enzyme production was natural gas (personal communication with Kløverpris, J. H., Novozymes A/S, February 2015), while the source of heat is unknown. Total primary energy input was therefore assumed to correspond to the reported input of fossil primary energy.
ii. The update of the Cellic CTec3 carbon footprint data from 8 to 5.5 kg CO_2_-eq. kg^−1^ of product corresponds to a reduction by 31%. An update of fossil primary energy input was assumed to follow the reduction of the carbon footprint, resulting in 69 MJ kg^−1^ of enzyme product.

The above assumptions and updated data were used to calculate the GHG emissions and primary energy balance for the off-site case, in order to enable a justified comparison to the integrated process designs.

##### Alternative enzyme data for sensitivity analyses

Impacts of off-site enzyme production could potentially be reduced, for instance by developing higher enzyme activity, enhancing energy efficiency or using a higher proportion of renewable energy. The size of potential improvements related to different measures and time horizons entails large uncertainties, and a detailed assessment was further complicated by scarce and aggregated data. In an attempt to relate to and discuss our results, we present GWP results for off-site enzyme production using the following alternative data and assumptions leading to various potential improvements.

For the sensitivity analysis, off-site enzyme dosage data were adapted from [7] (12.4 g enzyme cocktail kg^−1^ DM) and alternative GHG data for enzymes from [10] and [11] (16 and 2.3 kg CO_2_-eq. kg^−1^ enzyme, respectively). Using alternative GHG data for enzyme production required a different method to decide enzyme dosage: we assumed 65 FPU g^−1^ of whole, purchased enzyme preparation containing 10% enzyme protein according to [32]. Furthermore, we tested assumptions of a 50% increase in enzyme activity based on previous improvements from earlier generations of the Novozymes A/S Cellic CTec product (personal communication with Kløverpris, J. H., Novozymes A/S, February 2015). Lastly, an assumption of Swedish electricity mix for the production was tested to exemplify a scenario with a larger share of renewable electricity. For this purpose, a rough estimation of electricity use in off-site enzyme production was based on general data for industrial enzyme production [45]: electricity was estimated to contribute to roughly 40% of GHG emissions from the enzyme production. Replacing natural gas-based electricity [45] with the Swedish electricity mix was estimated to reduce GHG emissions by 70% kWh^−1^ electricity, thus reducing total GHG emissions from enzyme production by roughly one-third.

##### Electricity and fuels

The electricity and fuel data used in the calculations of GWP and primary energy input are presented in Table 1. Natural gas-based electricity was included in aggregated, off-site enzyme production data. Because of the aggregated nature of these data, combined with a lack of information or alternative data regarding a more detailed breakdown of primary energy and GWP, assumptions regarding electricity and fuels in off-site enzyme production were necessary. The procedure for such assumption is described in the “Enzyme and nutrients” section. For the integrated cases, assumptions regarding electricity and fuels are mainly based on the geographic location. As the proposed ethanol plant was assumed to be located in Sweden, electricity supply in case A, which is the only case requiring input of grid electricity for the bioethanol production, was assumed to be the average Swedish grid electricity. For the same reason exported electricity was assumed to substitute the Swedish electricity mix in all ISO calculations. In the sensitivity analysis, grid electricity was assumed to be natural gas-based electricity. Assuming competing interests for forest residues for biofuel and bio-based heat [70], lignin solid fuel was assumed to replace wood-based pellets. In the sensitivity analysis, lignin pellets were assumed to substitute hard coal for heating.

## Results and discussion

### Minimum ethanol selling price

Results for MESP in EUR L^−1^ ethanol were as follows: off-site 0.581, A 0.622, A+ 0.595, B 0.586 and B+ 0.568. Figure 3 shows the MESP of ethanol with off-site enzyme production, plotted as a function of enzyme price, and MESP for each of the integrated cases. In regard to MESP, case B+ was the most favourable. Furthermore, this was the only case with integrated enzyme production in which the MESP was lower than that in the off-site case with purchased enzymes. The off-site case, case B and case B+, result in MESPs below the estimated average ethanol selling price of 0.59 EUR L^−1^, thus resulting in positive annual cash flows (see Additional file 1).

In the off-site case, we calculated an approximate enzyme cost of 0.078 EUR L^−1^ ethanol (39 MSEK year^−1^ for the production of 6760 L ethanol h^−1^ for 8000 h year^−1^). In a previous statement from Novozymes A/S, the predecessor product to Cellic CTec3 was said to cost just under 0.5 USD gallon^−1^ lignocellulosic ethanol [71], corresponding to approximately 0.1 EUR L^−1^ (using 2010 exchange rates, 1 EUR ≈ 1.3 USD). Our cost estimate could thus be reasonably close to that of the more recent Cellic CTec3 enzyme product. In the study by Liu et al. [13], an enzyme cost of 0.5 USD gallon^−1^ ethanol from corn stover was the basis for enzyme case 3, which was the intermediate scenario investigated. Thus, the estimated cost of enzymes in the off-site case presented here appears to compare reasonably well with the previous studies and available information, though the raw material and enzyme dosage underlying the calculated costs in [71] are unknown.

Factors other than enzyme cost affect the MESP and economics of the systems studied. An example is the selling prices of co-products, though in this case annual cash flows show that ethanol is the most important source of income (see Additional file 1). The cash flows are calculated on an ethanol selling price of 0.59 EUR L^−1^ but actual market prices for ethanol vary over time. In Sweden, the yearly average consumer price of E85, an ethanol blend fuel of 15% gasoline, varied by 5% above and below the average price in the years 2010–2015 [72].

### Life-cycle assessment

#### Greenhouse gas emissions

**Fig. 4:**
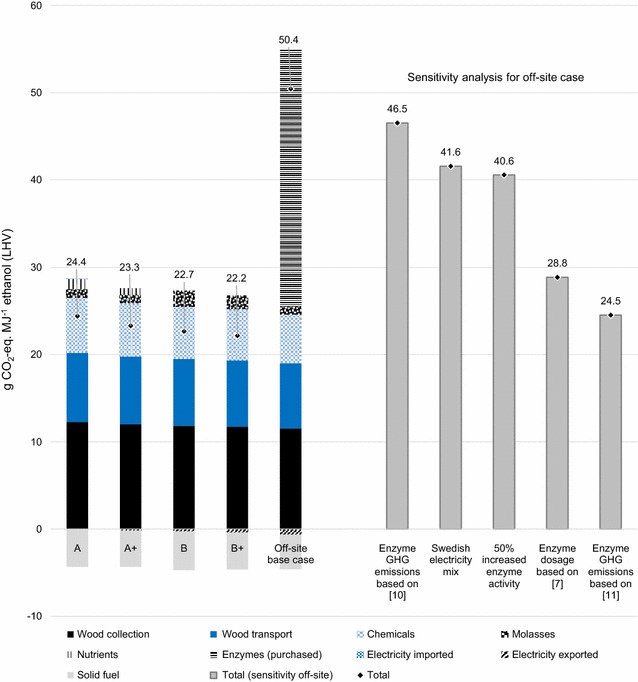
GHG emissions (expressed as GWP) for assessed ethanol production systems using the ISO method. Sensitivity analyses for off-site enzyme production, using alternative data and assumptions, are included. For illustration purposes, “Chemicals” include SO_2_, NH_3_, H_3_PO_4_, antifoam, (NH_4_)_2_HPO_4_ and MgSO_4_. “Nutrients” include soybean oil meal, (NH_4_)_2_SO_4_, KH_2_PO_4_ and FeSO_4_*7H_2_O

**Fig. 5:**
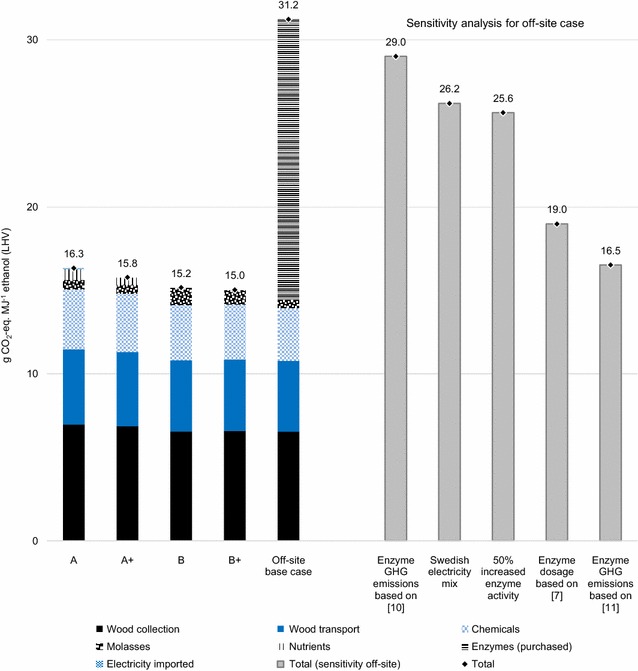
GHG emissions (expressed as GWP) for assessed ethanol production systems using the RED method. Sensitivity analyses for off-site enzyme production, using alternative data and assumptions, are included. For illustration purposes, “Chemicals” include SO_2_, NH_3_, H_3_PO_4_, antifoam, (NH_4_)_2_HPO_4_ and MgSO_4_. “Nutrients” include soybean oil meal, (NH_4_)_2_SO_4_, KH_2_PO_4_ and FeSO_4_*7H_2_O

The results for GWP of ethanol using the ISO and RED calculation methods are presented in Figs. 4 and 5, respectively. In the base case scenario, the off-site case resulted in significantly higher GWP than the integrated cases and case B+ showed the lowest GWP (Figs. 4, 5). The purchased enzymes contributed with 18 and 30 g CO_2_-eq. MJ^−1^ ethanol in the base case (RED and ISO methods, respectively). Since these results show that off-site enzyme production could be a major contributor to the GHG emissions of ethanol in the off-site case, alternative data and assumptions are tested in sensitivity analyses, where the GWP of off-site enzyme production was reduced by 13–88%. Implications to the comparison between off-site and integrated cases are illustrated in Figs. 4 and 5. It should also be noted that GWP results are generally lower using the RED method (Fig. 5) than the ISO method (Fig. 4), which is explained by the difference in LCA method choices.

With the GHG emissions value 16 kg CO_2_-eq. kg^−1^ enzyme protein from [10], which is based on Aspen modelling of an off-site scenario, the GWP of the off-site case was still higher than those in all other cases (Figs. 4, 5). Assuming 2.3 kg CO_2_-eq. kg^−1^ enzyme protein as in [11], the GWP of the off-site case was reduced significantly, and was roughly equal to the GWP of case A using both calculation methods. In [11], co-location of enzyme and ethanol production was assumed, using part of the hydrolysate from ethanol conversion from switchgrass to grow *T. reesei* for cellulase production. Thus, this scenario presents an on-site case, partly integrated with ethanol production.

Applying the enzyme dosage from [7] (12.4 g enzyme cocktail kg^−1^ DM) to the off-site case (which assumes a dosage of 30.4 g enzyme cocktail kg^−1^ DM) gave a 73% reduction of the base case enzyme GHG emissions (Figs. 4, 5). The total GWP of the off-site case was still higher than the GWP of the integrated cases, but the difference between them was reduced significantly. The dosage in [7] was based on a Novozymes A/S dosage data sheet for cellulose conversion in corn stover.

Assumptions of 50% increased enzyme activity and a Swedish electricity mix both reduced the GHG emissions from enzyme production by roughly 30%. If these improvements were assumed simultaneously, the GWP from purchased enzymes would be reduced by approximately half, leading to roughly a 30% reduction of the total GWP in the off-site case. Total GHG emissions were still higher in the off-site case than in cases with integrated enzyme and ethanol production (Figs. 4, 5).

Various input data available regarding the GHG emissions from off-site enzyme production [10, 11] indicate the significant uncertainties in the estimation of total emissions of ethanol. The dosage of purchased enzymes in lignocellulosic ethanol production was a significant uncertainty in assessing the total GHG emissions, as illustrated by adopting dosage data from [7]. The base case scenario illustrated the current state of enzyme production, using GHG emission data based on actual plant performance, and enzyme activity of a purchased cellulase cocktail as reported in the literature. The lower GWP results obtained when using data from [11] can be explained by the future and partly integrated scenario used in which enzyme activity is higher and GHG emissions are lower. On the other hand, enzyme dosage as calculated in [7] did not make any assumptions on future improvements, but nevertheless resulted in a significant reduction of the total GWP of the off-site case compared to the base case scenario.

It is thus difficult to draw general conclusions regarding both the current state of off-site enzyme production, as well as its future improvement potential. Nevertheless, the cases and results presented in this study indicate that an integrated process design could provide lower GHG emissions for lignocellulosic ethanol production. For instance, the replacement of natural gas-based electricity with renewable alternatives did lower the GHG emissions of off-site enzyme production, but based on the estimation made, the integrated cases result in even lower GHG emissions for ethanol (Figs. 4, 5). In order to improve the comparison between emerging integrated production designs and existing off-site enzyme production systems, further studies are, however, needed to provide a more detailed analysis of off-site enzyme production, including potential improvements.

The results for GWP were also tested with alternative assumptions for substituted products and changes in soil organic carbon (SOC) levels due to the recovery of logging residues in spruce stands. Table 2 shows that the ISO GWP results are sensitive to assumptions regarding substituted products. Assuming substitution of natural gas-based electricity decreases total GWP by 0–18% for different cases, and when solid fuel was assumed to replace coal for heating all cases resulted in negative GWP values. All cases were affected (except case A for substituted electricity) and the results of the comparison between off-site and integrated cases did not change significantly.

**Table 2:** Sensitivity analysis of ethanol GHG emissions (expressed as GWP)

g CO_2_-eq. MJ^−1^ ethanol (LHV)	ISO					RED				
	Off-site	A	A+	B	B+	Off-site	A	A+	B	B+
Base case^a^	50	24	23	23	22	31	16	16	15	15
SOC 2–3 rotations	78	53	52	51	50	47	33	32	31	31
SOC 1 rotation	111	89	86	85	84	66	53	52	50	50
SOC 20 years	700	710	700	670	670	500	510	500	480	480
Solid fuel substitutes coal	−21	−64	−62	−68	−64					
Electricity substitutes natural gas-based electricity^b^	44	24	22	20	18					

Results with alternative data and assumptions regarding soil organic carbon (SOC) and substituted products

^a^Base case includes only fossil GHG emissions, excluding changes in SOC levels

^b^In this scenario imported electricity in case A is also assumed to be natural gas-based

Including potential effects on SOC levels according to [58] significantly increased GHG emissions in all cases. Cases with integrated enzyme production use more feedstock per MJ ethanol produced, and were thus affected to a greater extent than the off-site case. Changes in SOC levels differ between the northern and southern Sweden, with biogenic CO_2_ emissions in the north of the country approximately twice the magnitude of those in the south in the long term. Here we chose an average case to illustrate the potential magnitude of the results for Swedish spruce residues. Because the degradation of biomass in the forest is time-dependent, results are affected by the time horizon chosen, where shorter time spans increase the impact of SOC changes significantly.

Assuming a long-term scenario of two to three rotation periods (231 and 240 years in southern and northern Sweden, respectively [58]) for changes in SOC, GHG emissions increased by approximately 54–126% using the ISO method and by 50–104% using the RED method. The off-site case still resulted in higher GWP than the integrated cases. Assuming one rotation period (77 and 120 years in southern and northern Sweden [58]), ISO GWP results were above or at the same level as the RED fossil fuel reference of 83.8 g CO_2_-eq. MJ^−1^, still with the off-site case causing the highest emissions. With a time horizon of only 20 years, all GWP results increased drastically, with results of up to 710 and 510 g CO_2_-eq. MJ^−1^ ethanol for the ISO and RED methods, respectively. On this assumption, the off-site case had a lower GWP than case A but higher than case B, with both calculation methods.

#### Primary energy balance

**Fig. 6:**
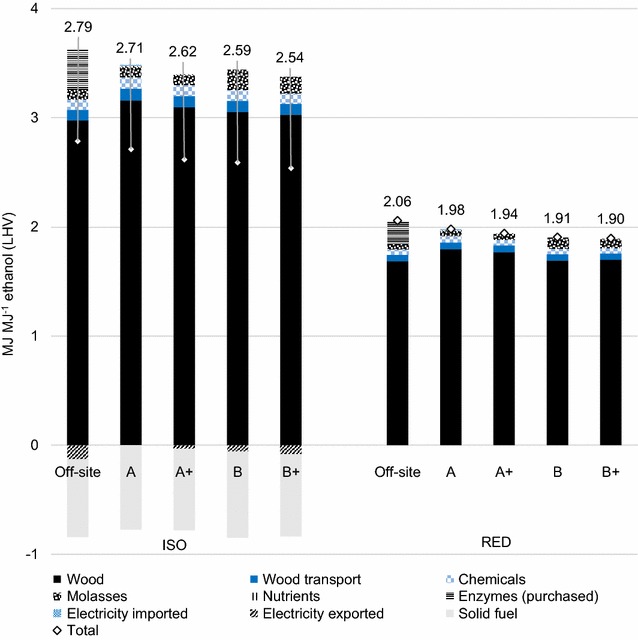
Primary energy balances for the ethanol production systems studied. Calculations according to the ISO method (*left*) and the RED method (*right*)

Case B+ showed the lowest primary energy input per MJ of ethanol, and the off-site case showed the highest (Fig. 6). Feedstock energy was the main contributor to primary energy input for all cases.

Using the ISO method and the RED method (in parentheses), primary energy efficiency, expressed as primary energy output over input, was as follows: off-site 36% (49%), A 37% (50%), A+ 38% (51%), B 39% (52%) and B+ 39% (53%). The efficiency was slightly lower in the off-site case, with 3% units between the highest and lowest result using the ISO method, and 6% units using the RED method.

The sensitivity of primary energy balances was tested in relation to dosage of purchased enzymes and lignin solid fuel substitution (Table 3). As feedstock was the main contribution to primary energy input, the parameters tested had limited impact on the results (4–10% change from the base case scenario). However, with alternative assumptions regarding enzyme dosage, the primary energy balance of the off-site case was approximately equal to that of case B+. In general, the difference between an integrated and an off-site approach regarding primary energy efficiency did not appear significant enough to justify drawing any definite conclusions, considering the aggregation and uncertainty of the input data.

**Table 3:** Sensitivity analysis of primary energy balances of the ethanol production systems studied

MJ primary energy MJ^−1^ ethanol	ISO					RED				
	Off-site	A	A+	B	B+	Off-site	A	A+	B	B+
Base case	2.8	2.7	2.6	2.6	2.5	2.0	2.0	1.9	1.9	1.9
Off-site enzyme activity +50%	2.7					2.0				
Enzyme dosage based on [7]	2.5					1.9				
Solid fuel substitutes coal	2.7	2.6	2.5	2.5	2.4					

Results for alternative data regarding purchased enzymes and substituted products

## Conclusions

This study assessed the economic and environmental aspects of integrated enzyme production in lignocellulosic ethanol production. An LCA compared the modelled integrated cases to an off-site case regarding emissions of GHG and primary energy balance, and a techno-economic analysis compared the cost performance.

The results show that a new integrated process design for ethanol and enzyme production could lower GHG emissions from lignocellulosic ethanol, compared to existing ethanol production with purchased enzymes. Regarding primary energy efficiency, no significant difference was identified in this study. The LCA results are sensitive to assumptions regarding the production and utilization of purchased enzymes, e.g. dosage needed and energy sources utilized in production. Although the GHG emissions of off-site enzyme production could be lowered, for instance by replacing fossil energy sources, drawbacks are the refining and stabilization processes not needed in an integrated process design. It is possible that separated processes could provide other benefits that have not been investigated here, where focus was on the potential of an integrated process. Thus, updated and more detailed studies are required for off-site enzyme production systems.

Within LCA, assumptions regarding crediting co-products and system expansion, especially the inclusion of effects on soil organic carbon levels from logging residue recovery, were crucial to the end result for the GHG performance of ethanol. However, in most cases, such assumptions did not significantly affect the comparison of the integrated production approaches to the off-site case with off-site enzyme production.

The economic feasibility of integrating enzyme production in the lignocellulosic ethanol process depends on the price of the full-scale commercial preparation of cellulase enzyme. In this study, off-site production resulted in the lowest MESP with the exception of one integrated case (case B+). This implies that integrated enzyme production can potentially be an alternative strategy considering process economics, provided that higher enzyme productivity and yield can be achieved than those presented in the laboratory trials.

To strengthen the comparison between off-site and integrated production, more detailed and updated data for off-site enzyme production are needed, as also further upscaling and implementation of both off-site and integrated processes.

## Additional files

**Additional file 1.** Mass balances, annual costs and revenues, and techno-economic details for the ethanol production systems studied. The file contains the mass balances and the economic data that were used to produce the results of this study. The file also contains annual cash balances for the ethanol production systems studied, providing further information on the implications of the results in the techno-economic analysis, and a process flow diagram and additional details for one of the studied systems.

**Additional file 2.** Aspen Plus data for the results of mass and energy balances. The overall process is shown in the first Excel sheet, and the hierarchies (PRETREAT, YC-E-SSF, DISTILL, EVP-SEP, DRYING, COMBUST) are in the subsequent sheets.

**Additional file 3.** Aspen Process Economic Analyzer data for the equipment cost and the total direct cost (in SEK). The quoted items (e.g. steam pretreatment unit) are not included in the list, as we do not have permission to present the quoted prices in details.

## Abbreviations

CEF: cellulase enzyme fermentation
DM: dry matter
FCI: fixed capital investment
FPU: filter paper unit
GHG: greenhouse gas
GWP: global warming potential
LCA: life-cycle assessment
MESP: minimum ethanol selling price
RED: renewable energy directive
SOC: soil organic carbon
SSF: simultaneous saccharification and fermentation
WCI: working capital investment
