# Supplementary material for: Integrating enzyme fermentation in lignocellulosic ethanol production: life-cycle assessment and techno-economic analysis
Source: Biotechnol Biofuels. 2017 Feb 23;10:51. doi: 10.1186/s13068-017-0733-0 (PMC5324314; doi:10.1186/s13068-017-0733-0)
Supplement: Supplementary file 1 — Additional file 1. Mass balances, annual costs and revenues, and techno-economic details for the ethanol production systems studied. The file contains the mass balances and the economic data that were used to produce the results of this study. The file also contains annual cash balances for the ethanol production systems studied, providing further information on the implications of the results in the techno-economic analysis, and a process flow diagram and additional details for one of the studied systems. [file 13068_2017_733_MOESM1_ESM.docx]

# Additional file 1. Mass balances, annual costs and revenues, and techno-economic details for the ethanol production systems studied.

## Mass and energy balances

The sulphur dioxide has the same mass flow in all the cases, since the raw material input is identical (Table 1). The ammonia demand is greater in the integrated CEF cases than in the off-site case, as extra ammonia is required in the CEF, which is directly proportional to the total mass flow fed to the CEF. The phosphoric acid is added in the WWT, and its mass flow is equal in all the cases. The amount of antifoam added to the ethanol fermentation is based on experimental data, and is directly proportional to the ethanol produced. The antifoam requirement of the CEF is assumed to be equal to that of ethanol fermentation. The mass flows of (NH_4_)_2_HPO_4_ and MgSO_4_ depend on the mass flow fed to the SSF, and therefore they are constant. The molasses addition does not differ significantly in the off-site case and cases A and A+, since the molasses is required only for the yeast fermentation. In cases B and B+ the CEF also utilizes molasses, and due to the higher activity yield, in B+ less molasses is added than in B. In A and A+ the mass flows of soybean oil meal, (NH_4_)_2_SO_4_, KH_2_PO_4_ and FeSO_4_*7H_2_O are directly proportional to the total mass flow fed to the CEF.

Among the scenarios electricity is imported only in case A: the total mass flow fed to the CEF and consequently the power consumption of the compressor are the highest in this scenario. The demand for cooling water differs because of the heat generated in the fermentations: it is lowest in the off-site case due to the lack of the CEF. In cases A and B the amount of cooling water used is higher than in A+ and B+, respectively, since at lower activity yield more carbohydrate is metabolised to reach the given activity level, that is, more heat is released. The demand for process water varies within a narrow range, and refers to the make-up water used in the CHP, as the diluting water added to the pretreated slurry before filter pressing is recycled from the evaporation. The make-up water is used to generate steam which is directly injected in the pretreatment. However, vapours from the dryer are also recycled to the pretreatment. The more solid fuel is produced, the more vapours the dryer generates, and consequently the less make-up water is required by the CHP for steam generation for the pretreatment.

The enthalpy flow of solid fuel is higher in the case of molasses addition in the CEF (case B and B+). The off-site case gives the highest ethanol production, and the more carbohydrate is consumed from the liquid fraction of the diluted slurry in the CEF, the less ethanol is produced. Carbon dioxide is evolved in yeast cultivation, SSF and CEF, and there is a higher total mass flow of carbon dioxide in the integrated cases than in the off-site case. The electricity export depends on the power consumption of the compressor of the CEF, which is lower at a lower total mass flow fed to the CEF. The mass flow of cleaned water from the aerobic treatment is lower in the CEF cases, as more water is recycled from the evaporation to dilute the pretreated slurry compared to in the off-site case.

Table 1. Material and energy flows of the modelled ethanol production cases.

| Material and energy flows of the modelled cases. | | Off-site | A | A+ | B | B+ |
| --- | --- | --- | --- | --- | --- | --- |
| Spruce dry matter | kg h^-1^ | 25000 | 25000 | 25000 | 25000 | 25000 |
| SO_2_ | kg h^-1^ | 641 | 641 | 641 | 641 | 641 |
| NH_3_ (25 %) | kg h^-1^ | 641 | 699 | 679 | 668 | 659 |
| H_3_PO_4_ (50 %) | kg h^-1^ | 5.6 | 5.6 | 5.6 | 5.6 | 5.6 |
| Antifoam | kg h^-1^ | 13 | 24 | 24 | 24 | 25 |
| (NH_4_)_2_HPO_4_ | kg h^-1^ | 74 | 74 | 74 | 74 | 74 |
| MgSO_4_ | kg h^-1^ | 3.7 | 3.7 | 3.7 | 3.7 | 3.7 |
| Molasses | kg h^-1^ | 885 | 886 | 887 | 1807 | 1497 |
| Soybean oil meal | kg h^-1^ | 0 | 97 | 63 | 0 | 0 |
| (NH_4_)_2_SO_4_ | kg h^-1^ | 0 | 29 | 19 | 0 | 0 |
| KH_2_PO_4_ | kg h^-1^ | 0 | 14 | 9 | 0 | 0 |
| FeSO_4_*7H_2_O | kg h^-1^ | 0 | 0.19 | 0.13 | 0 | 0 |
| Enzyme preparation | kg h^-1^ | 761 | 0 | 0 | 0 | 0 |
| Electricity imported | MW | 0 | 0.2 | 0 | 0 | 0 |
| Cooling water | m^3^ h^-1^ | 1961 | 2079 | 2041 | 2086 | 2048 |
| Process water | m^3^ h^-1^ | 12.5 | 12.4 | 12.5 | 12.2 | 12.3 |
| Ethanol | L h^-1^ | 6758 | 6360 | 6493 | 6584 | 6640 |
| Solid fuel | MW (LHV) | 28.0 | 28.5 | 28.1 | 30.2 | 29.1 |
| Carbon dioxide | kg h^-1^ | 5731 | 5906 | 5847 | 6085 | 5967 |
| Electricity exported | MW | 2.4 | 0 | 0.6 | 1.1 | 1.5 |
| Water from aerobic treatment | m^3^ h^-1^ | 29 | 27 | 27 | 27 | 27 |

Carbon source A: pretreated liquid fraction, B: pretreated liquid fraction and molasses; +: 1.5-fold specific activity.

## Techno-economic analysis

The data used to calculate minimum ethanol selling prices (MESP) are presented here in Table 2. Annual cash flows (Table 3) illustrate the distribution of costs and revenues for the ethanol production systems studied.

Table 2. Data for costs and prices.

| Input or product | Purchase or selling price, or cost | |  |
| --- | --- | --- | --- |
| *Raw material* |  |  | |
| Spruce | 60.2 | EUR dry tonne^-1^ | |
| *Chemicals* |  |  | |
| SO_2_ | 0.16 | EUR kg^-1^ | |
| NH_3_ (25 %) | 0.2 | EUR kg^-1^ | |
| H_3_PO_4_ (50 %) | 0.5 | EUR kg^-1^ | |
| Antifoam | 2.2 | EUR kg^-1^ | |
| (NH_4_)_2_HPO_4_ | 0.16 | EUR kg^-1^ | |
| MgSO_4_ | 0.47 | EUR kg^-1^ | |
| Molasses | 0.1 | EUR kg^-1^ | |
| Soybean oil meal | 0.16 | EUR kg^-1^ | |
| (NH_4_)_2_SO_4_ | 0.1 | EUR kg^-1^ | |
| KH_2_PO_4_ | 0.1 | EUR kg^-1^ | |
| FeSO_4_*7H_2_O | 0.1 | EUR kg^-1^ | |
| *Utilities* |  |  | |
| Electricity | 48.4 | EUR MWh^-1^ | |
| Cooling water | 0.01 | EUR m^-3^ | |
| Process water | 0.15 | EUR m^-3^ | |
| *Other costs* |  |  | |
| Labour | 64516 | EUR employee^-1^ year^-1^ | |
| Insurance | 1 | % of annual fixed capital | |
| Maintenance | 2 | % of annual fixed capital | |
| *Co-products* |  |  | |
| Pellets | 124 | EUR dry tonne^-1^ | |
| Electricity, spot price | 38 | EUR MWh^-1^ | |
| Electricity certificate | 22 | EUR MWh^-1^ | |
| Carbon dioxide CO_2_ | 3.2 | EUR tonne^-1^ | |

Purchase prices of raw material, nutrients, chemicals, utilities, costs of labour, insurance, maintenance, and selling prices of co-products [1,2].

Table 3. Annual costs, revenues and profit of the proposed ethanol plant in million euros (MEUR).

|  | Off-site | A | A+ | B | B+ |
| --- | --- | --- | --- | --- | --- |
| Annual cost (MEUR) |  |  |  |  |  |
| Raw material | 10.7 | 10.7 | 10.7 | 10.7 | 10.7 |
| Capital | 16.9 | 19.7 | 19.4 | 19.1 | 18.8 |
| Chemicals | 3.04 | 3.48 | 3.41 | 4.09 | 3.81 |
| Enzymes | 4.2 | - | - | - | - |
| Utilities | 0.25 | 0.35 | 0.26 | 0.27 | 0.26 |
| Other | 2.30 | 2.39 | 2.38 | 2.37 | 2.35 |
| Total | 37.4 | 36.6 | 36.0 | 36.6 | 35.9 |
| Annual income (MEUR) |  |  |  |  |  |
| Ethanol | 31.9 | 30.1 | 30.8 | 31.2 | 31.4 |
| Co-products except electricity | 4.85 | 4.94 | 4.87 | 5.22 | 5.03 |
| Electricity | 1.13 | 0 | 0.28 | 0.51 | 0.71 |
| Total | 38.0 | 35.1 | 35.9 | 36.9 | 37.2 |
| Annual profit (MEUR) | 0.56 | -1.56 | -0.17 | 0.31 | 1.27 |

All costs and revenues are calculated at an ethanol selling price of 0.59 EUR L^-1^ (5.5 SEK L^-1^). Carbon source A: pretreated liquid fraction, B: pretreated liquid fraction and molasses; +: 1.5-fold specific activity.

## Further details and data

Scenario A was chosen arbitrarily to present some techno-economic details (Fig. 1 and Tables 4 and 5). The results of mass and energy balances are reported in the Excel file of Aspen Plus data (Additional file 2). The overall process is shown in the first Excel sheet, and the hierarchies (PRETREAT, YC-E-SSF, DISTILL, EVP-SEP, DRYING, COMBUST) are in the subsequent sheets. The equipment cost and the total direct cost (in SEK) are listed in the Excel file of Aspen Process Economic Analyzer data (Additional file 3). The quoted items (e.g. steam pretreatment unit) are not included in the list, as we do not have permission to present the quoted prices in details.

Fig. 1. Mass flows (in kg h^-1^) of the Scenario A. LP: low-pressure (4 bar), HP: high-pressure (20 bar), WWT: wastewater treatment, EtOH: ethanol.

Table 4. Heating and cooling duties of the process areas (in kW) in Scenario A.

|  | Pretreatment | Fermentation | Distillation | Evaporation | Drying | Total |
| --- | --- | --- | --- | --- | --- | --- |
| Heating with process stream | 0 | 0 | 10196 | 45960 | 164 | 56320 |
| Cooling with process stream | -6862 | 0 | -259 | -48733 | -466 | -56320 |
| Heating with steam from CHP | 0 | 0 | 6537 | 9311 | 3247 | 19095 |
| Cooling with utility | -1093 | -7962 | -4431 | -14887 | 0 | -28373 |
| Total cooling duty | -7955 | -7962 | -4690 | -63620 | -466 | -84693 |
| Total heating duty | 0 | 0 | 16733 | 55271 | 3411 | 75415 |

The combined heat and power production (CHP) generates 6120 kW electricity and 19095 kW heat used for indirect heating in the process. The direct steam usage of the pretreatment is not included in the table (the mass flows of direct steam injection are shown in Figure 1).

Table 5. Breakdown of the total capital investment cost in MEUR in Scenario A.

|  | MEUR |
| --- | --- |
| Raw material handling | 1.26 |
| Pretreatment | 14.46 |
| Cellulase enzyme fermentation | 11.07 |
| Yeast cultivation and SSF | 15.09 |
| Distillation | 5.91 |
| Stillage separation & evaporation | 9.31 |
| Drying & pellet production | 5.41 |
| Combined heat and power production | 19.62 |
| Storage | 4.28 |
| Heat exchanger network | 2.26 |
| Anaerobic digestion | 1.76 |
| Total direct cost | 90.42 |
| Total indirect cost | 85.02 |
| Fixed capital investment | 175.44 |
| Working capital | 5.74 |
| Total capital investment | 181.18 |

SSF: simultaneous saccharification and fermentation.

## References

[1] Sassner, P., Galbe, M., Zacchi, G. (2008) Techno-economic evaluation of bioethanol production from three different lignocellulosic materials. *Biomass Bioenergy*. 32:422-30.

[2] Sassner, P., Zacchi, G. (2008) Integration options for high energy efficiency and improved economics in a wood-to-ethanol process. *Biotechnol Biofuels*. 1:4.
